# Questions worth asking for futures worth making: an effectual approach

**DOI:** 10.1007/s11187-023-00747-5

**Published:** 2023-04-21

**Authors:** Saras Sarasvathy

**Affiliations:** Paul Hammaker Professor of Business Administration, University of Virginia, The Darden School, Charlottesville, VA USA

**Keywords:** Effectuation, Predictive reasoning, Entrepreneurial expertise, Cocreation, Problem of plenty, Loss aversion, Self-selection, Stakeholders, Negative contingencies, Teleology, D81, D91, E71, I25, L26, M13

## Abstract

It is not unusual in the psychology and economics of entrepreneurship to focus on decision models based on predictive reasoning that explain outcomes such as venture creation (at the micro level), firm performance (meso level), or job creation (macro level). However, in this article, derived from the literature on entrepreneurial expertise, I argue for an embrace of uncertainty, where outcomes are not only unknown, but unknowable, hence undermining predictive criteria for actions and decisions. By focusing on principles and processes that do not entail predictive reasoning, effectuation offers both practical guidance for acting in the face of multiple uncertainties and novel research questions not yet examined through the lens of the entrepreneurial method. Specifically, I offer five possible new ventures for future research built on the five principles of effectuation. These pertain to new futures worth making, without prescribing or predicting what those would, could, or should be.

## Abstract (plain language summary)

How can effectual entrepreneurs not only build valuable ventures, but also take on existential threats and build futures no one has dreamed of yet? Entrepreneurship education is becoming increasingly important and widespread in the twenty-first century. However, the content of most education programs draw rather haphazardly from research pieces in psychology and economics or from popular business books written by entrepreneurs, business gurus, and bloggers. In contrast, a cohesive stream of over a hundred academic articles, under the name “effectuation,” has sought to spell out a more rigorous basis for understanding how entrepreneurs act on, react to, and interact with uncertain situations involving everything from the decision to leave a job to start a new venture to building and growing that venture. Effectuation specifies ways of acting that do not require entrepreneurs to predict and place bets on the future. Instead, effectuators work with things within their control and with others who want to work with them, to cocreate new ventures and even new markets and new futures. Effectual action thus makes entrepreneurship a method, not only to create jobs and economic opportunities but also to help tackle large societal problems.

## Introduction

Jamsetji Tata, the founder of the Tata Iron and Steel Company of India died in 1904 and did not live to see the company formed in 1907 (Fraser, 1919). But he had begun working on the venture in 1882, when he had chanced upon a report on iron deposits in India by German geologist Ritter Von Schwartz. It is not easy to explain how or why a man who had already built a textile mill and luxury hotel would invest the last two decades of his life in an unlikely enterprise in iron and steel. Note that this story is not atypical of other entrepreneurs, from Josiah Wedgwood in the eighteenth century to Elon Musk and Jack Ma today.

The story of entrepreneurship is filled with chance encounters, goose chases, moments of heroism intermixed with follies and epic fails. It is also a story of hard work, work to build relationships inside and outside existing networks and organizing these into valuable enterprises. Neither the success after the fact nor the wisdom to start the venture in the first place can be justified within familiar frames of predictive reasoning.

For that, we need an effectual approach (Sarasvathy, 2001, 2008).

An effectual approach embraces uncertainty (Knight, 1921; Townsend et al., 2018). More importantly, it turns our attention to the fact that uncertainty is not a problem to be overcome. Instead, uncertainty is both ingredient and outcome of our best efforts to overcome it. Consider the current moment in history.

Thanks to science, humanity has come to a point where we increasingly hold the power to literally shape our future, without understanding what we want that future to be. We are concurrently faced today with unprecedented threats from climate change and migration. But the age of pandemics and nuclear weapons is not behind us. Yet at the societal level, we continue to proceed as though we can all agree a priori on particular futures embodied in specific goals with dedicated budgets of human and financial resources before we act.

In fact, while we have more ways at our disposal to achieve what we want, we also have more ways to disagree about what we could, should, and will actually want. On the one hand, we need to find alternatives to the pretense that our understanding of what is worth wanting can come from ancient texts or instincts shaped through biological evolution or random preferences that we have little control over. On the other hand, given multiple uncertainties, we cannot rely on rationality as the sole or even primary guide to our aspirations.

Scientific approaches uncover reality as it is, at present and in the past, as well as what it could be in the future. But in addition to these, we need a way to act on this reality to transform it into what we want it to be without having to predict or concur ahead of time on what that might turn out to be.

Let us consider a recent example to see why knowledge from science and history are not enough for the task ahead. The twentieth century saw an unprecedented increase in the near-eradication of dozens of diseases such as small pox, polio, and measles through vaccination (Wolfe & Sharp, 2002). Concurrently, references to vaccine hesitancy in medical publications was close to zero through the first decade of the twenty-first century. Yet in ways that no one could or did predict, resistance to vaccination shot up exponentially in the past decade, notably before 2019 (Dubé et al., 2021; Larson et al., 2022). Consequently, in the face of a vaccine to effectively combat the global pandemic of COVID 19, 77% of the US population expressed some degree of hesitancy and the World Health Organization listed vaccine hesitancy as one of the top 10 threats to global health. Even more unpredictable was the phenomenon of thousands of unvaccinated Germans migrating to Paraguay in the last couple of years of the pandemic, many of them illegally (Pereyra, 2022).

Scientific education, history, democracy, even prosperity, are not enough to lead us to consensus about what is worth doing and becoming, as persons and societies. Even when we agree on goals worth achieving, we discover difficulties prioritizing and choosing between them (Burkeman, 2021; Tversky et al., 1990). Worse still, even when we actually achieve some of those goals, they turn out to have unintended consequences leading to further uncertainties (Kasser, 2016; Staw, 1984).

Entrepreneurs learn this while acting within contexts of at least three kinds of intertwined uncertainties: Not only do we not know how to achieve our goals, we often do not know what goals we want to achieve. Add to that the problem of too much, rather than too little information, and our knowledge can disorient rather than guide us.

The question of what is worth wanting has mostly been deemed outside the scope of science. In the sciences, questions of why and what to value are transformed into questions of how, with answers formulated in terms of deterministic or probabilistic predictions (Hull, 2010; Poincaré & Maitland, 2003). Physics seeks to answer how the universe came into being and the laws through which it functions, but not why. Biology explains how species evolve without explaining what makes life worth living. Economics simply takes preferences and utility functions as given rather than seeking to explain them.

Entrepreneurship cannot abdicate the question of what is worth wanting, since it is a method of constructing and effectuating exactly that (Sarasvathy & Venkataraman, 2011). In other words, entrepreneurship is a method to cocreate purposes and futures worth achieving. It does not prescribe what is worth doing, like ethics or moral philosophy might do. It also does not offer explanations for why we find something more or less worth pursuing, as politics and psychology might do. Instead, entrepreneurship offers a procedure for shaping purposes without prescribing them a priori or simply explaining them after the fact. I will now briefly summarize what we have learned about that procedure in the past two decades.

## The principles and processes of effectuation: navigating as well as shaping the prediction control space

Effectual entrepreneurship offers five principles for cocreating futures in the face of:

- Knightian uncertainty—the future is not only unknown but unknowable (Knight, 1921);
- Goal ambiguity—we may not always know what we want (March, 1978); and
- Isotropy—it is not clear which information is relevant or irrelevant to decision and action (Fodor, 1987).

At the heart of effectuation is a recasting of the relationship between prediction and control. Conventional views, including the scientific approach cast prediction as leading to control (Wiltbank et al., 2006). Over the past two decades, hundreds of studies have examined the experiences of entrepreneurs who have learned to separate prediction from control (Alsos et al., 2020). In other words, control can be used as a strategy to transform uncertainty into goals worth achieving and worlds worth making.

**Fig. 1:**
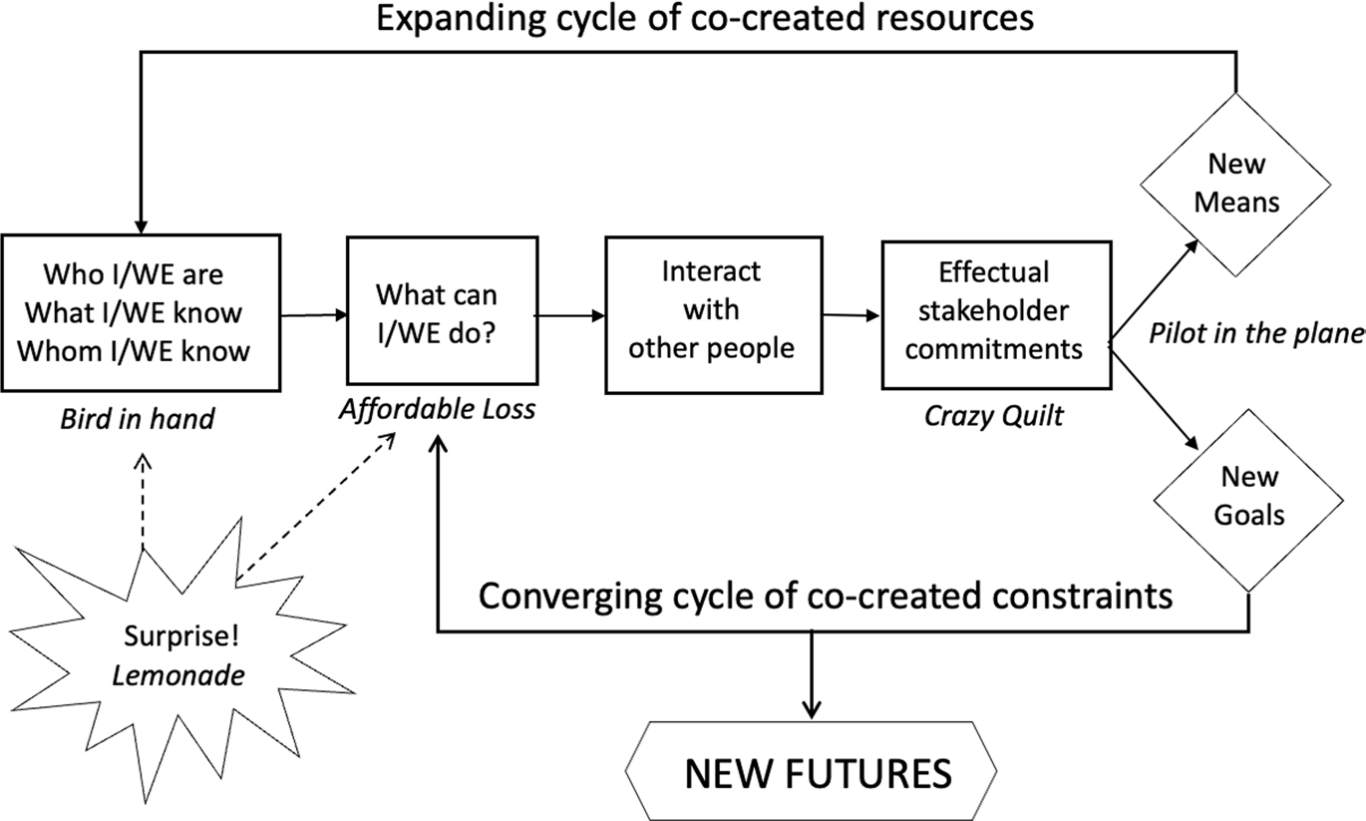
The effectual process (note: the five principles of effectuation are depicted in italics)

Through a dynamic process (as illustrated in Fig. 1) that incorporates the five principles of effectuation, entrepreneurs routinely work with things within their control not only to navigate but also to shape and cocreate the prediction-control (PC) space (Sarasvathy & Dew, 2005).

It is easy to mistake effectuation as a form of bootstrapping or trial and error process involving gut-feeling or intuition. But there is a deliberate logic to effectual action anchored in the separation of prediction and control. At every point in the PC space and every moment in the effectual process, we can uncover this logic simply by examining whether and how much we are relying on predictive information. This logic is teachable and learnable and comprises entrepreneurial expertise acquired through deliberate practice of the simplest of interactions between two people, namely the “ask” (Sarasvathy, 2022a).

**Fig. 2:**
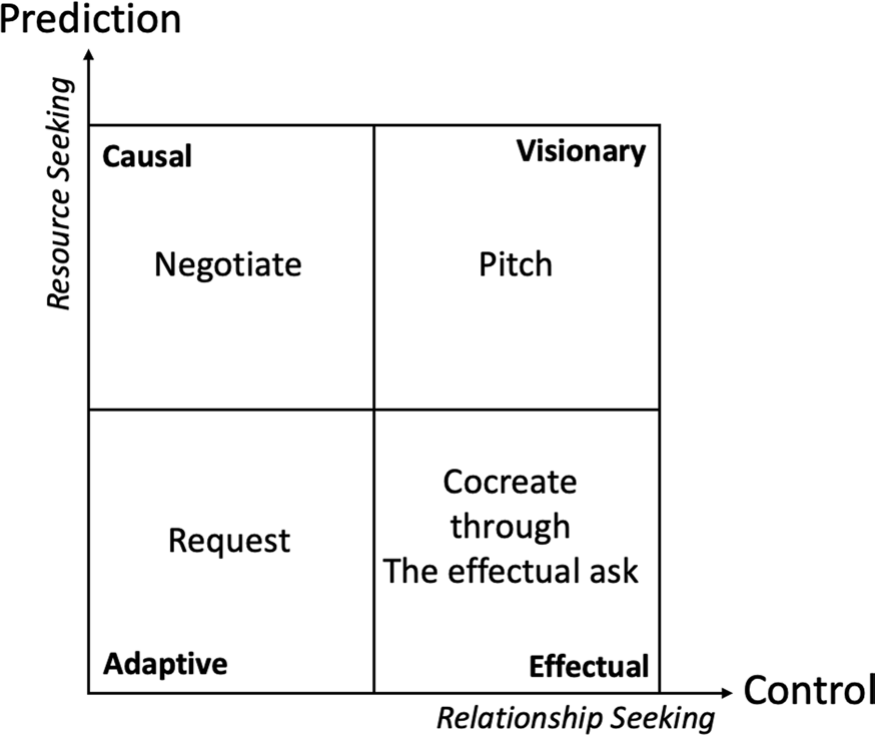
The prediction control (PC) space

The ask is a pre-exchange phenomenon. It consists of at least two persons, an asker and an askee, and a situation transformable into something of value. The ask itself can be taxonomized through the PC space as illustrated in Fig. 2. When the asker is clear about what s/he wants and from whom, she comes up with a pitch. However, note that the pitch is but one kind of ask, appropriate to the visionary quadrant of the PC space. It can be contrasted with an adaptive ask consisting in requests for advice, feedback, and help of various kinds. Asks can also be causal, seeking out predictive information specific to particular venture goals and targeted stakeholders, such as market research leading to customer acquisition or financial forecasting leading to investor acquisition.

Finally, asks can be effectual, involving cocreative conversations with anyone and everyone, inside and outside of one’s networks. When these conversations result in commitments, they can then be fashioned into unexpected, innovative and valuable goals that kickstart the shaping of new futures.

The more familiar story of prediction can be found in science, whether publicly or privately funded, leading to new technologies that in turn lead to new futures, whether intended or otherwise. Often these futures require regulatory responses as the technologies get embodied in products, ventures, opportunities, and threats.

The role of entrepreneurship within this story is not as clear. Entrepreneurs are sometimes confused with the invisible hand of markets and at other times with monopolistic practices of large corporations, or the heroic soap opera of passionate individuals (Sarasvathy, 2022c). Effectuation spotlights a more nuanced reality consisting in varieties of individuals and organizations coming together in partial and dynamic coalitions between self-selected stakeholders to cocreate futures.

In the past two decades, evidence has been proffered for effectual action leading not only to for-profit and nonprofit ventures (Johannisson, 2018; Read et al., 2016; Yusuf & Sloan, 2015), but also to polycentric governance systems (Sarasvathy & Ramesh, 2019), art movements (Callander, 2019; Olive-Tomas & Harmeling, 2020), disaster relief (Nelson & Lima, 2020), ways to combat climate change (Urban, 2018) and foster entrepreneurship within native communities (Murphy et al., 2020).

### *Effectual action leading to new futures*

Let us now consider categories of futures that can be made through effectual action. A non-exhaustive list may include the following:

- Futures involving existential threats
- Futures of work, leisure, and income
- Futures of markets, states, and money
- Futures of freedom, fairness, and faith
- Futures of mind, attention, and compassion

Even a cursory look at the list shows that from physics to politics, uncertainty in all its varieties and dimensions seems to permeate our lives.

Existential threats loom large (Ord, 2020). In addition to climate change and nuclear war, we have to contend with new technologies and the new teleologies they enable. Consider just two: artificial intelligence (AI) and synthetic biology (synbio). When we think about AI, we mostly think about projects such as AlphaZero, the analysis of big data or driverless cars. Or killer robots. But how about AI-enabled best friends for our children? Or all our daily experiences mediated through wearable AI?

Synbio offers the prospect of combating climate change through animal-free meat. But it also opens the door to reengineering our very identity as a species. High school students around the world are engaged in synbio today because the cost of creating new species of viruses and other life forms has decreased a thousand-fold in recent years. As we successfully extend access to higher education beyond 15% of humanity, the frontiers of knowledge grow, and fundamental changes increase in frequency and impact.

In addition to direct outcomes from technology that challenge preconceived notions of what might count as the good life and even what it means to be human, daily lives of ordinary human beings and hence socio-political realities and contours of the economy all seem to be in flux. Take our work lives, for example. In developed countries, as David Graeber (2018) argues in “Bullshit jobs,” it is already unclear whether the jobs we work at produce any tangible value. Even a less provocative view suggests that specialized competence related to division of labor as argued by Adam Smith is less the source of our incomes today than careers that call for creativity, caring, and compassion. However, most of these may not produce growing incomes of the kind enabled by industrialized production. At the same time, it might now be feasible to provide Universal Basic Income (Bidadanure, 2019) to many if not all, thereby increasing choices over leisure and work for entire populations, raising the question of what is worth doing with one’s time.

At the societal level, familiar institutions that have worked well in the past, whether free markets or liberal democracies are similarly in flux. Threats and opportunities arise not only from geopolitics, but also from social media and the blockchain. Cryptocurrencies may be overhyped but smart contracts and distributed autonomous organizations offer ways to go beyond rearranging markets and governments to new architectures for human interactions, whether resulting in exchanges in new marketplaces or governance in new organizational forms and institutions (Sarasvathy, 2022b).

In the meanwhile, deeper understandings of the brain and mental health, through advances in neuroscience, reopen questions of freedom and justice as well as the constitution of equitable societies and their allocation of blame and responsibility. These call into question taken for granted assumptions about morality and merit. In fact, an increasing awareness of randomness and luck in ability and opportunity combined with the possibility of artificial enhancement of both, lead us to question the wisdom of meritocracy itself as a value worth pursuing (Arrow et al., 2018; Markovits, 2019).

In sum, simply assuming prediction leads to control is grossly inadequate. Yet current literatures in innovation and entrepreneurship confuse hypothesis testing with market building (Zellweger & Zenger, 2022). Turning to data to elicit predictions for the future and then placing bets on them confounds polling with governance and investments with innovation.

Polling can provide useful information but what to do with that information, still requires individual ingenuity, crafty negotiations, creative competition as well as cocreational coalitions. Similarly, investments often hinder valuable innovations and discourage as well as enable entrepreneurship (Aldrich & Ruef, 2017). As Hayek argued, idiosyncratic individuals and even ignorance itself are necessary ingredients for the creative flourishing of civilizations (Hayek, 1977). Moreover, innovations in teleology are as important as innovations in technology.

Given that the effectual process does cocreate new teleologies as well as new ventures and markets that embody these, it would be interesting to consider a research agenda relating effectuation to new futures worth making.

## New intellectual ventures for new futures

When we run out of interesting new ideas and theories, we start offering questions and research agendas instead Without ducking that possibility, let me outline a set of five questions based on each of the five principles of effectuation. The questions can be tackled at multiple levels of analyses. I do not mean these to be problems to be solved. Rather I offer them as intellectual ventures for researchers as well as educators, policy makers, and entrepreneurs to effectuate.

### The bird-in-hand principle: what if the only scarce resource is the human lifespan?

It is customary to think of the allocation of scarce resources to unlimited wants as the central problem of economics (Samuelson, 1948). However, we could also invert the problem and consider a world in which the only scarce resource is human time and mind-space.

One measure consists in 80,000 h (40 years at 40 h a week, 50 weeks a year) as the time available to most human beings for productive work (Todd, 2016). The problem then becomes one of figuring out what to do with those 80,000 h. Or with the entire lifespan, estimated at around 4,000 weeks (Burkeman, 2021). Note that even though this resource is limited at the individual level, it tends to be within one’s control, very much a part of one’s bird-in-hand. Additionally, at the level of society, this scarcity at the individual level could itself turn into a problem of plenty.

Furthermore, this problem of plenty is not hypothetical, even at the individual level. I encounter it with young people both in prosperous and penurious places in the world. Let me offer examples:

- Example 1. *I’m an only child. My parents’ house is fully paid for and will eventually come to me. I have a great education and am in good health. I see some of my classmates have such passion for what they want to do. I don’t seem to have such passion. How do I decide what to do with my life?*
- Example 2. *I grew up in the slums. I took up music and mixed martial arts as a way to survive here. Recently I was offered a fellowship to visit Europe and was stunned by the doors that opened for me. I would like to change the circumstances I grew up in. But I would also like to go back to Europe and help my family from there. I’m not sure what I should do.*

Again, this issue is not limited to the level of the individual. Founders of NGOs return from places like Haiti, disillusioned by the fact that free money and goods that come in as aid kill local enterprise (Moyo, 2009; Schuller, 2012). And governments grapple with the possibility of job losses to AI and struggle to understand the pros and cons of concepts like Universal Basic Income (Friedman, 2013; Van Parijs, 2013).

As we save more children from infant mortality and push access to higher education beyond 15% of the population (Roser & Ortiz-Ospina, 2016), problems of plenty begin to appear. Careful consideration of these raises the specter that problems of plenty may be even more dystopic than problems of penury (Ord, 2020). However, my aim here is neither to peddle utopias nor dystopias, but to argue for the need for practical and reasonable methods to deal with the problem of what is worth doing—under circumstances of abundance as well as those of scarcity.

### The affordable loss principle: how can we educate to move beyond loss aversion and myopic gain?

On the one hand, both fear and greed are primal drives that can and have been harnessed to engender working institutions of freedom and prosperity. On the other hand, they are engines of devastation, fostering injustice and perpetuating poverty. It is necessary to acknowledge and attend to loss aversion and the myopia of greed, maybe even leverage their importance to survival. But it is equally important to design education that helps us move beyond them, thereby enabling individuals and societies to move beyond instinct toward informed choice and considered judgment.

Both loss aversion and myopic gain are consequences of the evolutionary biology of fear. Jervis (1992, p. 187) provides a concise yet telling synopsis of prospect theory:

*In summary, the theory argues that people tend to be risk-averse for gains (this was generally known before) but simultaneously to be risk-acceptant for losses (this was the surprise). People are loss-averse in the sense that losses loom larger than the corresponding gains. Losing ten dollars, for example, annoys us more than gaining ten dollars gratifies us. What is peculiar about this is that, contrary to most versions of expected utility theory, the reference point—usually the status quo—is crucial (Tversky & Kahneman, 1986). More than the hope of gains, the specter of losses activates, energizes, and drives actors, producing great (and often misguided) efforts that risk—and frequently lead to—greater losses.*

Loss aversion paradoxically leads to higher levels of risking losses! Finance is strewn with evidence for this paradox in practice. In fact, we have reified the correlations between risk and return uncovered in empirical studies in finance into a maxim of life that high risk is a necessary condition for high return.

But is this conclusion justified? Of course, when we examine ventures with high returns, we will find some correlations to high levels of investments, whether from public or private funding sources. However, we can also find cases of high returns that did not require highly risky investments or even high levels of investment at all. Take, for example, the data point that the majority of ventures that go public are not funded by venture capital (Gompers & Lerner, 2001). Companies like Microsoft and eBay are cases in point. The so-called unicorns also abound outside the technology sector—Starbucks, Kinkos, Spanx—the list could go on. Thus, both within and outside the technology sector, a majority of large and enduring high-performance firms did not require high risk, or even high levels of funding.

Yet, both academic literature and media stories are acutely biased toward funded ventures and mostly silent on unfunded ones. We need to investigate ALL ventures with all kinds of funding sources including those with very little or no outside funding.

In fact, we need to develop a much deeper understanding of risk and return as multi-dimensional constructs that change over time. Moreover, the different dimensions and dynamics of both risk and return are subject to uncertainties and ambiguities of various kinds that can be traded off to reach the frontier of low risk-high return. The affordable loss principle provides a useful window on this possibility. It focuses attention not on the upside but on the downside. It provides the comfort of keeping the downside within one’s control. Yet cues in a different sort of discussion on the upside that goes beyond more is better to uncover what may be worth striving for even if one loses what one is willing to invest in any given enterprise.

At the macro level, the affordable loss principle urges the public square to examine and commit to both what is affordable and what is NOT at different moments and contexts. Additionally, it also requires policy makers to contemplate the incorporation of failures into their decision-making. Finally, this view combines with bird-in-hand to highlight the idea that resources are scarce only if you ignore the fact that every resource you need is likely within the affordable loss level of someone else in the world. In this sense, both the problem of plenty and tools to overcome loss aversion require an understanding of working with self-selected stakeholders.

### The crazy quilt principle: how can self-selection channel merit and reduce violence?

At the end of my classes, I often ask students if they are bullish on the human race. Even the most optimistic tend to estimate the odds only at slightly above 50%. I then bring them statistics from Hans Rosling (2018) and Steven Pinker (2012), both of which make a compelling case for progress, highlight the outsized role of violence in impeding that progress, as well as the downward trends in violence. This has led me to start thinking about work to be done at the confluence of entrepreneurship and violence. How can effectuation help people *self-select* out of violence?

Mechanisms of selection dominate the study of decision-making. One reason for that is the phenomenal success of predictive approaches in science. Another is the effectiveness of ecological competition and natural selection in evolution. Based on a predictive approach, we try to incentivize behavior through selective carrots and sticks. And based on an evolutionary perspective, we embrace competition not only in the marketplace, but in life, pruning the population out of good schools, good jobs and good healthcare in the name of selective merit.

It would be interesting to see whether and how we can channel merit effectually, by offering the option of shaping new futures with uncertain consequences rather than expectations of rewards and punishments based on predetermined objectives. Another way to think about this is in terms of getting people to see themselves as partners in uncertainty and not purveyors and recipients of carrots and sticks (Underhill, 2016). Or winners and losers in games of chance, while pretending those are games of merit.

My linking violence and merit might at first seem incongruous. Even more difficult to see might be the link to entrepreneurship. Yet, consider the example of the Bard Prison Initiative (BPI) in the US founded in 1999 by undergraduates at a private university in response to the government canceling funding for college education within prisons. The recidivism rate for graduates of BPI is 20% compared to 96% otherwise. In addition to education, there is also historical evidence for what Steven Pinker calls “gentle commerce” in the reduction of violence and war (Pinker, 2012).

Effectual entrepreneurship research can add to these by offering details of interactions between self-selecting individuals and small groups that can transform zero-sum games into structures with positive sums. Even in the face of negative contingencies.

### The lemonade principle: what does it take to build institutions that foster cocreative responses to negative contingencies?

When something bad happens, we move immediately to damage control and begin learning aversive lessons so we can prevent that occurring in the future. Understanding of natural evolution provides a compelling case for adaptive responses based on the predictive brain (Clark, 2013). Yet it is possible to overlearn the lessons from evolution and undervalue conscious cocreation.

Lewontin (1991) pointed to this difference between evolution and history:

*Like the House of Lords that destroyed its own power to limit the political development of Britain in the successive Reform Acts to which it assented, so the genes, in making possible the development of human consciousness, have surrendered their power both to determine the individual and its environment.*

In other words, it is up to us to develop institutions that foster freedom and fairness in a world filled with contingencies.

I grew up in India with stories of the struggle for independence from the British, with recurring parables related to Mahatma Gandhi, starting with his being thrown out of the first-class compartment of the train on his way to Pretoria in South Africa. Remember this was not the Gandhi of the loin cloth. This was the young “gentleman,” a freshly minted barrister from London. His nonstandard response to what was a standard experience of injustice at that time puzzled me. It made me ashamed of my own instincts to simply give in and move over to the van compartment. It was clear I was no hero. Later I had the opportunity to read about the incident in Gandhi (1983, p. 135), where he describes that night he spent in the cold waiting room of Maritzburg railway station. It was bitterly cold because of the high altitude and also because Gandhi’s coat was in his luggage that the officials had taken away.

*My over-coat was in my luggage, but I did not dare to ask for it lest I should be insulted again, so I sat and shivered. There was no light in the room. A passenger came in at about midnight and possibly wanted to talk to me. But I was in no mood to talk.*

*I began to think of my duty. Should I fight for my rights or go back to India, or should I go on to Pretoria without minding the insults, and return to India after finishing the case? It would be cowardice to run back to India without fulfilling my obligation.*

This time, I began to understand something about courage. Framing it as a choice between being a hero or not, was very different from framing it as one between being a coward or not. Furthermore, when I was originally told the story in heroic terms of a man whose response to injustice was to start the Satyagraha movement, the task seemed overwhelming and impossible, a task for a superhero. But the real story consists in the experiences and events that occurred between that cold night in Maritzburg and the twenty years of life in South Africa that followed. The “making” of Gandhi consisted in conversations and interactions with others, which then resulted in the forging of ingenious mechanisms and institutions of nonviolent protest, which in turn propelled the “making” of a new future for his country of origin.

One of the biggest hindrances to good decision-making and the making of institutions based on good decision-making is outcome bias (Baron & Hershey, 1988). Because we bind ourselves to prespecified goals without examining how those goals came to be in the first place, we often become blind to the cocreative potential of negative contingencies. Instead, we ossify our preventive impulses into heroic myths as well as bureaucratic institutions rather than explore new ways of being and becoming that these contingencies open up for us.

Exploring these new ways entails acute psychological discomfort – giving up what we considered worth striving for. We tend to experience this discomfort in terms of “abandonment” of things we have come to hold dear, even our sense of who we are. In turn this prevents us from tapping into one of the most important tools of valuable innovation – uncertainty itself.

As we take up the question of how we can go beyond adaptive responses to effectually transformative and cocreative ones, we can begin tackling the task of making entrepreneurial societies in conjunction with democratic and scientific ones.

### The pilot-in-the-plane principle: when is entrepreneurship a particularly useful alternative to democracy and science, even as it builds on both?

In the past two hundred years of the histories of nation-states, we have seen a variety of combinations of capitalism and democracy as well as non-capitalism and non-democracy. Through almost all of these, the power of the scientific method has waxed more than it has waned. Acknowledging but setting aside for the moment valid critiques of the scientific method or the very notion of “method” itself, I would urge both the need and potential for research, education, practice and policy built around the entrepreneurial method. In particular, we need to approach entrepreneurship not merely as a subset of market economics or democratic politics, but as a powerful alternative that can at the same time build on and transcend both these more familiar forces. Furthermore, the entrepreneurial method is not subsumed under the scientific method either. It is distinct from it even when it works in tandem to it.

I am often asked how big hairy problems can be solved with effectuation. In response, at the risk of Herbert Simon rolling over in his grave, I argue that an effectual approach requires us to reconsider the very notion of problem-solving. Some problems can indeed be “solved” through technological innovation. Others need to be dissolved, transcended and even used as resources for teleological innovation. Work on this front is just beginning. But the first step is to identify and acknowledge the standing of entrepreneurship alongside democracy and science as a sculptural force of human history.

Effectuation does not offer a utopic vision. By dint of being non-predictive and non-teleological, it cannot. Instead, it offers a practical method to concurrently forge vision and value through entrepreneurial action, interaction, and reaction. There are many ways to experience and shape the flow of history. So far we have learned what we can do when we include the invisible hand of the market and the voices of the many to work with the iron fist of the state. In other words, we have already seen that it is possible to move beyond the grunt of the ape within our throats.^1^ Let us add to that a cocreative embrace of uncertainty. Our task ahead is to combine chance and loss with conversations and commitments as inputs into the making of valuable persons and societies without waiting to have those specified in advance.

Kiran Mazumdar Shaw learned to do this after being disappointed in her dream to become a brew master. The beer industry in India did not exactly welcome her into the profession. And when an accidental introduction through a mutual acquaintance led to a request to help source biopharmaceuticals from India, her initial response was to turn it down. With a bit of coaxing, she reluctantly agreed to begin with the manufacture of enzymes, the kind she knew something about from brewing. Unlike Jamsetji Tata who did not live to see his iron and steel company, Kiran Mazumdar Shaw has lived to build and run the four billion dollar venture that is Biocon.

## Conclusion

The point, however, is not the value of any given venture, successful or otherwise, but the shaping of what may be worth valuing that we cannot even dream of today. I would like to end with a quote from Jacqueline Novogratz, co-founder of Acumen Fund:

*…if you would’ve asked me or any of my co-founders in 1986, when women had just gotten the right to open a bank account without their husband’s signature, if you had said to us that in 30 years we wouldn’t just be improving women’s economic condition which was our mission, but that a young woman in the next generation would be running the financial system and that Rwanda would have more women parliamentarians than any country on the planet, I’m not sure we would have believed you. Maybe our dreams were too small.*

## Data Availability

All data and materials used in this article support their published claims and comply with field standards.

## Footnotes

1 Inspired by John Ciardi’s poem “Possibilities.”
